# Patient perception of service quality to preanesthetic oral examination: a cross-sectional study using the SERVQUAL model

**DOI:** 10.1186/s12903-024-03853-2

**Published:** 2024-01-22

**Authors:** Ju-Hui Wu, Kun-Tsung Lee, Kuang-I Cheng, Je-Kang Du, Chen-Yi Lee

**Affiliations:** 1 Department of Oral Hygiene, College of Dental Medicine, Kaohsiung Medical University, No. 100, Shih-Chuan 1st Road, Kaohsiung, 80708 Taiwan; 2 Department of Dentistry, Kaohsiung Medical University Hospital, Kaohsiung, Taiwan; 3 Department of Anesthesiology, Kaohsiung Medical University Hospital, Kaohsiung, Taiwan; 4 Department of Anesthesiology, Faculty of Medicine, College of Medicine, Kaohsiung Medical University, Kaohsiung, Taiwan; 5 School of Dentistry, College of Dental Medicine, Kaohsiung Medical University, Kaohsiung, Taiwan; 6 Department of Medical Research, Kaohsiung Medical University Hospital, Kaohsiung, Taiwan

**Keywords:** Interdisciplinary cooperation, Patient safety, Perioperative dental injury, Quality improvement, Service quality

## Abstract

**Background:**

A phase-III interdisciplinary quality improvement program, the preanesthetic oral examination (PAOE), was implemented as a new program in an academic medical center to prevent perioperative dental injuries. This study was aimed at surveying the perceived service quality and satisfaction of patients who had undergone PAOE based on the SERVQUAL model.

**Methods:**

This cross-sectional survey was conducted at the Kaohsiung Medical University Hospital using convenience sampling. Patients referred for PAOE (PAOE group) and those who had voluntarily availed dental services (control group) were recruited. A modified SERVQUAL questionnaire was used to assess the perceived service quality and patient satisfaction with dental services. Cronbach’s alpha for SERVQUAL was 0.861.

**Results:**

We enrolled 286 (68.8%) and 130 (31.2%) participants in the PAOE and control groups, respectively. The path analysis revealed that the PAOE group scored lower in dimensions of reliability (β = -0.074, *P* = 0.003), responsiveness (β = -0.148, *P* = 0.006), and empathy (β = -0.140, *P* = 0.011). Furthermore, reliability (β = 0.655, *P* < 0.001) and responsiveness (β = 0.147, *P* = 0.008) showed a direct effect on patient satisfaction. Overall, participants were highly satisfied with the dental services.

**Conclusions:**

The PAOE group showed lower satisfaction and perceived quality of dental services compared to the control group. Although implementing an interdisciplinary program reduces the perceived service quality, its influence is limited. Employing an interdisciplinary teamwork is a win–win strategy encouraged to improve patient safety and reduce malpractice claims. Future suggestions should focus on establishing waiting times that are considered reasonable by patients. Patient-centered education related to the risk of perioperative dental injuries should be provided, and awareness of oral conditions for patient safety should be improved. Moreover, interprofessional education in continuous and undergraduate programs is necessary to improve professional quality.

**Supplementary Information:**

The online version contains supplementary material available at 10.1186/s12903-024-03853-2.

## Background

Perioperative dental damage is a common anesthesia-related adverse event responsible for many malpractice claims against anesthesiologists [1–5]. The incidence of dental trauma after anesthesia varies with the study design. Prospective studies (2.23–38.6%) tend to report significantly higher rates of dental injury compared to retrospective studies (0.02–0.7%) [3, 6, 7], owing to possible selection bias and underreported injuries in retrospective studies [7]. Laryngoscopes are commonly used in perioperative intubations to lift the epiglottis for proper visualization of the larynx. The use of the maxillary teeth as a fulcrum, and of excessive pressure, may lead to dental injuries when the laryngoscope is employed [7]. After surgery, patients with poor dental conditions may accidentally injure their teeth before they fully recover from anesthesia.

For risk avoidance, associated staff education and department policy changes to optimize the management of dental trauma have become increasingly crucial to address the entire preoperative evaluation process, which is essential for improving medical quality and patient safety [3, 8, 9]. Prevention of dental damage starts with the identification of risk factors. All patients with vulnerable teeth should be evaluated by a dentist for remedial or restorative dental work to prevent damage preoperatively. Securing a loose tooth or mouth guard is a cautious measure to prevent aspiration and aid tooth retrieval in the case of dislodgment [9]. Although a consensus on the preoperative protocol for cooperation between anesthetists and dentists is required, different institutions have adopted different strategies to prevent dental injuries and promote patient safety.

At an academic medical center in Taiwan, the first interdisciplinary quality improvement program for reducing dental injury rates was executed in 2011. It has been proven to be efficient [3]. After implementing a two-phase quality improvement program, the incidence of dental injury reduced significantly from 0.108 to 0.051% and remained at a low level of 0.009% [3]. Therefore, preanesthetic oral examination (PAOE) has been extended as a new program in the hospital. Although these improvements in reducing dental injury rates have benefited the hospital system, patient experiences have not been adequately studied. Patient satisfaction and service quality related to preoperative assessment clinics is generally high [10–12]. Communication of information is the most positive component related to satisfaction, while waiting time is the most negative one [11, 12]. The SERVQUAL model has revealed a similar finding [10]. However, these preoperative assessments have not included oral examinations.

The aim of the present study was to detect relationships between perceived service quality and the satisfaction of patients who had undergone a phase-III quality improvement program, i.e., PAOE, based on the SERVQUAL model. We hypothesized that implementing the new program would change the perceptions of service quality and satisfaction in dental patients, whether or not they receive anesthesia. Exploring the SERVQUAL dimensions immediately after dental services could more accurately elucidate the elements that contribute to patient satisfaction in the interdisciplinary program.

## Methods

### Interdisciplinary quality improvement program

At the Kaohsiung Medical University (KMU) Hospital, the Quality Improvement Program was conducted from February 1, 2010, to July 31, 2011. Phase I of the program was conducted from August 1, 2011, to July 31, 2012, while phase II was initiated on August 1, 2012. Kuo et al. in 2016 reported the detailed contents of the two-phase program [3]. In November 2018, phase III was implemented as a new program applied to all patients who required general anesthesia. PAOE was conducted in the family dentistry department to improve safety and service quality for all eligible patients.

As the standard procedure, the surgeon referred the patient to a dentist for an oral examination before inducing general anesthesia for an elective surgical operation. A visiting staff member explained the need for an oral examination, then a detailed examination was performed by an intern, the results were double-checked by a resident, and finally triple-checked by the visiting staff member. If tooth mobility was detected, the dentist managed the pathologically mobile teeth with wire fixation or extraction to minimize the dental injury associated with anesthesia preoperatively. The goals of preoperative dental evaluation were to assess the patient’s oral status, provide oral hygiene instructions, and reduce the risk of dental damage. After oral examination, recording of the degree of tooth mobility was uploaded to the Hospital Information System for healthcare providers to check and alert critical information.

According to a statistical report from the KMU Hospital’s Patient Safety Bulletin, the average incidence rate of dental injury between November 2016 and October 2018 was 0.0255%. After the PAOE in November 2018, the average incidence rate of tooth damage between November 2018 and July 2020 was 0.0144%.

### Study design

This was a cross-sectional study using convenience sampling. The survey was conducted from August 2020 to April 2021. We enrolled patients referred from other departments for PAOE (PAOE group) and those who attended the family dentistry department for dental services (control group). A trained researcher explained the study purpose to eligible patients, and informed consent was obtained before delivering the questionnaire. The study protocol was approved by the Human Experiment and Ethics Committee of the Chung-Ho Memorial Hospital, KMU (KMUHIRB-E(II)-20,200,166).

### Instruments

The survey comprised three parts: demographic information (including sex, age, educational level, residence, and job), questions regarding the participants’ perspectives on dental services, and the perceived service quality items adapted from the SERVQUAL survey developed by Parasuraman et al. [13]. Additionally, the clinical characteristics of patients in the PAOE group (including the referral department, surgical history, tooth mobility, and tooth fixation) were collected from their medical records.

#### Measuring perspectives on dental services

Previous studies adopted “loyalty” as a construct, which included patient satisfaction, intention to recommend, and intention to return [14–16]. Considering the insignificance of the “intention to return” in the PAOE group, questions regarding perspectives on dental service were modified to include only overall satisfaction with hospital dental services and intention to recommend the service to others. Additionally, a question specific to patients receiving anesthesia regarding their attitude towards the necessity of PAOE was added. All answers were measured on a seven-point Likert scale from 1 (strongly disagree) to 7 (strongly agree).

#### Measuring perceived service quality

SERVQUAL was designed to measure consumer quality perceptions of services using 22 items across five dimensions, including tangibles (physical facilities, equipment, and appearance of personnel; four items), reliability (dependability with respect to timeliness and accuracy; five items), responsiveness (willingness to help customers and prompt service; four items), assurance (courtesy and inspiring trust and confidence; four items), and empathy (individualized consideration of a patient’s welfare; five items). In its original format, SERVQUAL measures the service–quality gap between patient expectations and perceptions. We measured only patient perceptions. We measured the service quality on a seven-point scale from 1 (strongly disagree) to 7 (strongly agree), with nine reverse-scored items. The score for each dimension was the mean value of the corresponding item scores. SERVQUAL has been widely used in many studies in Taiwan [15, 17–20]. In the present study, Cronbach’s alpha of the SERVQUAL scale was 0.861, indicating good internal consistency reliability.

### Statistical analysis

Questionnaires with incomplete or neglected reverse items were excluded from the statistical analysis. To further differentiate the perspectives on dental services between PAOE and control groups, the descriptions of each item were classified as “agree,” “non-committal or disagree,” and “no response.” “Agree” was rated 5–7 on the Likert scale; “non-committal or disagree” was rated 1–4; “no response” was considered as a missing value. The chi-square test was used to compare sociodemographic variables and perspectives on dental services between the two groups. The independent *t*-test was used to compare the scores in the five SERVQUAL dimensions. Statistical analyses were performed using Statistical Package for the Social Sciences (SPSS, version 20).

In order to examine the hypothesized relationships among the patient types, sociodemographic variables, five dimensions of SERVQUAL, and patient satisfaction, a path analysis model was developed and tested using AMOS 26. Rigorous evaluation criteria were adopted to ensure an adequate model fit. A χ^2^ test was chosen as the statistical test for the model fit (α = 0.05). As this test is sensitive to minor deviations in the model fit in large samples, comparative fit index (CFI), Tucker-Lewis index (TLI), standardized root mean squared residual (SRMR), and root mean square error of approximation (RMSEA) were used to evaluate the model fit. The following cutoff values were used to establish an adequate fit: CFI ≥ 0.95, TLI ≥ 0.95, SRMR < 0.08, and RMSEA < 0.06 [21].

## Results

### Sample characteristics

We included 416 valid questionnaires: 286 (68.8%) for the PAOE group and 130 (31.2%) for the control group. Respondents included 260 (62.5%) women and 156 (37.5%) men. The most prevalent age group was 40–49 years (27.4%), followed by 50–59 years (24.5%) and 20–29 years (21.4%). Most (61.5%) respondents had completed college or higher education, were residents of the Kaohsiung City (84.1%), and were employed (79.7%; Table 1). The comparisons between the groups revealed that the control group was significantly younger in age, had higher education levels, and had a lower percentage of employment (Table 1).

**Table 1:** Patient characteristics (*n* = 416)

Variables	N (%)			χ^2^	*P*
	Total	Patient type			
		PAOE (*n* = 286)	Control (*n* = 130)		
Sex					
Male	156(37.5)	108(37.8)	82(63.1)	0.027	0.870
Female	260(62.5)	178(62.2)	48(36.9)		
Age group (years)					
20–29	89(21.4)	50(17.5)	39(30.0)	12.553	0.014*
30–39	78(18.8)	51(17.8)	27(20.8)		
40–49	114(27.4)	87(30.4)	27(20.8)		
50–59	102(24.5)	77(26.9)	25(19.2)		
60–64	33(7.9)	21(7.3)	12(9.2)		
Education level					
College or higher	256(61.5)	159(56.0)	97(74.6)	13.945	< 0.001***
Senior/vocational high school	129(31.0)	100(35.2)	29(22.3)		
Junior high school or lower	29(7.0)	25(8.8)	4(3.1)		
Resident in Kaohsiung					
Yes	350(84.1)	245(85.7)	105(80.8)	1.604	0.205
No	66(15.9)	41(14.3)	25(19.2)		
Job					
Employed	318(79.7)	235(85.1)	83(67.5)	23.484	< 0.001***
Unemployed	55(13.8)	33(12.0)	22(17.9)		
Student	26(6.5)	8(2.9)	18(14.6)		

PAOE, preanesthetic oral examination; *: *P* < 0.05; ***: *P* < 0.001

### Clinical characteristics of patients in the PAOE group

Table 2 showed the clinical characteristics of patients in the PAOE group. The most frequent referral department was otolaryngology (29.7%), followed by gynecology and obstetrics (15.0%). Most (71.0%) participants were undergoing surgery for the first time at the KMU hospital. The oral examination revealed tooth mobility in 38 (13.3%) participants and tooth fixation in 24 (8.4%) participants.

**Table 2:** Clinical characteristics of participants in the preanesthetic oral examination group (*n* = 286)

Variables	Valid responses (n)	%
Referral department		
Otolaryngology	85	29.7
Orthopedics	29	10.1
Gynecology and obstetrics	43	15.0
Ophthalmology	18	6.3
Plastic surgery	28	9.8
Others	83	29.0
Surgery history at the KMU hospital		
First time	203	71.0
Not the first time	73	25.5
Canceled a surgery	10	3.5
Tooth mobility		
N	248	86.7
Y	38	13.3
Tooth fixation		
N	262	91.6
Y	24	8.4

KMU, Kaohsiung Medical University

### Perspectives on dental services

Overall, the participants were satisfied with the service in the family dentistry department (mean score: 6.24 ± 1.093) in both PAOE (mean score: 6.17 ± 1.208) and control (mean score: 6.39 ± 0.792) groups. Participants were willing to recommend the service (mean score: 6.16 ± 1.266) in both PAOE (mean score: 6.05 ± 1.400) and control (mean score: 6.37 ± 0.899) groups. Table 3 shows the results of transforming the scores of these items and combining them with the missing-value analysis. Compared to the control group, significantly more patients in the PAOE group answered “non-committal or disagree” or did not answer the question. Regarding the necessity of performing an oral examination before anesthesia, 240 (83.9%) participants answered “agree” (mean score: 6.15 ± 1.294); 28 (9.8%) participants answered “non-committal or disagree”; 18 (6.3%) participants did not answer.

**Table 3:** Comparing the perspectives on dental service by patient type

Perspectives	N (%)			χ^2^	*P*
	Total	Patient type			
		PAOE (*n* = 286)	Control (*n* = 130)		
Satisfaction					
“Agree”	365 (87.5)	237 (82.9)	127 (97.7)	18.463	< 0.001***
“Non-committal” or “disagree”	32 (7.7)	29 (10.1)	3 (2.3)		
No response	20 (4.8)	20 (7.0)	0 (0.0)		
Recommendation					
“Agree”	353 (84.9)	230 (80.4)	123 (94.6)	15.353	< 0.001***
“Non-committal” or “disagree”	46 (11.1)	39 (13.6)	7 (5.4)		
No response	17 (4.1)	17 (5.9)	0 (0.0)		
Necessity					
“Agree”	--	240(83.9)	--	--	--
“Non-committal” or “disagree”	--	28(9.8)	--		
No response	--	18(6.3)	--		

PAOE, preanesthetic oral examination; ***: *P* < 0.001

### Perceived service quality

Regarding the five dimensions of perceived service quality, tangibles, reliability, responsiveness, assurance, and empathy scored a total of 25.28, 31.93, 23.40, 25.37, and 30.75, respectively, and a mean of 6.32, 6.39, 5.85, 6.34, and 6.15, respectively. Table 4 shows the comparisons between PAOE and control groups. The PAOE group scored significantly lower in reliability, responsiveness, assurance, and empathy than the control group. In relation to sociodemographic characteristics, the younger age groups scored significantly higher in responsiveness and empathy; those with college or a higher education level scored significantly higher in responsiveness and empathy; and student status was significantly more associated with responsiveness and empathy (see Appendix). Sex and residence were of no significance in relation to the five perceived service quality dimensions.

**Table 4:** Comparing the perceived service quality by patient type

Variables	Patient type (M ± SD)		t	*P*
	PAOE (*n* = 286)	Control (*n* = 130)		
Tangibles (4–28)	25.07 ± 3.44	25.74 ± 2.85	-1.920	0.056
Reliability (5–35)	31.47 ± 4.42	32.92 ± 3.15	-3.783	< 0.001***
Responsiveness (4–28)	22.45 ± 7.00	25.48 ± 4.48	-5.240	< 0.001***
Assurance (4–28)	25.05 ± 4.31	26.05 ± 2.57	-2.918	0.004**
Empathy (5–35)	29.81 ± 7.56	32.82 ± 4.61	-4.946	< 0.001***

PAOE, preanesthetic oral examination; **: *P* < 0.01; ***: *P* < 0.001

### Path-analysis model

**Fig. 1:**
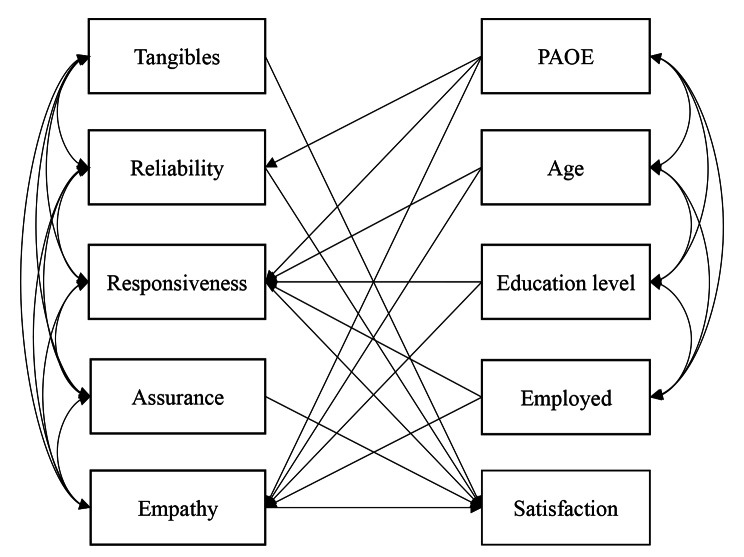
Theoretical framework of the study. A proposed path model predicting patient satisfaction PAOE, preanesthetic oral examination

**Fig. 2:**
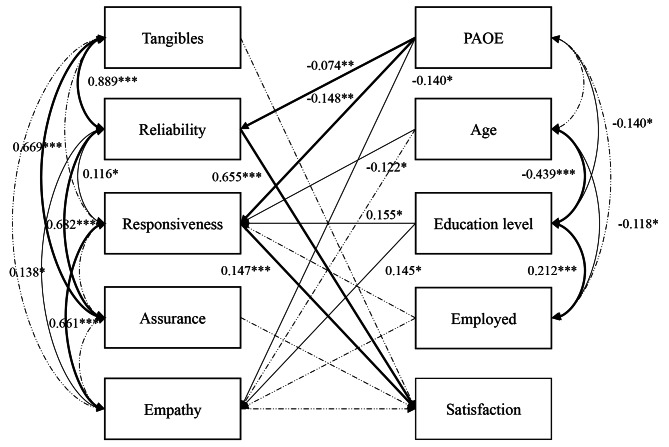
Path-analysis model relating service quality, patient type, and sociodemographic variables to patient satisfaction Standardized path coefficients are presented Non-significant paths are represented by dashed lines The significant paths with *P* < 0.01 are in bold Significance: *P < 0.05; **P < 0.01; ***P < 0.001 PAOE, preanesthetic oral examination

Figure 1 illustrates a proposed path model based on the literature review and the results of univariate analyses in this study. Participants who studied mostly at the medical university near the hospital with student status were excluded from the data analysis. Figure 2 illustrates the path-analysis model. The model fit to the data was satisfactory with the following values: χ^2^ = 19.765; *df* = 15; *P* = 0.181; SRMR = 0.038; RMSEA = 0.031, 90% confidence interval = 0.000, 0.065; CFI = 0.996 and TLI = 0.989. The model revealed that the PAOE group scored lower in reliability (β = -0.074, *P* = 0.003), responsiveness (β = -0.148, *P* = 0.006), and empathy (β = -0.140, *P* = 0.011) compared to the control group. Furthermore, reliability (β = 0.655, *P* < 0.001) and responsiveness (β = 0.147, *P* = 0.008) directly affected the patient satisfaction. Regarding the influences of sociodemographic variables, the older age group scored lower in responsiveness (β = -0.122, *P* = 0.038), whereas the group with a higher education level scored higher in responsiveness (β = 0.155, *P* = 0.011). The PAOE group was lower in education level. Hence, age, education level, and employment showed significant interrelationships between each other. Finally, the five dimensions of perceived service quality exhibited significant interrelationships.

## Discussion

In the present study, a detailed oral examination was performed by a dental team, including the assessment of tooth mobility. The tooth mobility and fixation rates were 13.3% and 8.4%, respectively. The posterior teeth with grade-I mobility were not fixed because of the unaffected pathology relative to the anterior teeth with grade-I mobility; therefore, they had a lower fixation rate. Moreover, POAE group in the phase III program showed a higher mobility rate than the pre-operative patients in phases I and II (7.53% and 9.74%, respectively) [3]. The difference between Phase III and Phase I or Phase II is that a qualified dental team surveyed the oral condition of the participant before surgery. Therefore, this result might be explained by the thorough examination conducted by the qualified dental team.

In the present study, patients in the PAOE group scored significantly lower in most dimensions of SERVQUAL compared to the control group. Moreover, they reported significantly lower satisfaction compared to the control group. In the path-analysis model, the five dimensions of SERVQUAL were interrelated. The patient type directly affected the reliability, responsiveness, and empathy scores; age directly affected the responsiveness score; educational level directly affected the responsiveness score and empathy scores; and the reliability and responsiveness scores were significant indicators affecting patient satisfaction (Fig. 2). The interrelationships between PAOE, age, education level, and employment indicated that patients who required general anesthesia were older and with a relatively lower education than the general dental patients. Therefore, they tended to possess lower health literacy [22]. The reliability scale indicates the service quality dimension representing timeliness and accuracy. A lower score indicates that the interdisciplinary quality improvement program increased the length of hospital stay of patients in the PAOE group, possibly leading to reduced patient satisfaction. Moreover, the responsiveness scale indicates the dimensions related to information and communication. PAOE is a new program; therefore, most patients may have insufficient knowledge about its importance, as demonstrated by the lower agreement of the necessity to perform an oral examination before anesthesia in the present study, this is consistent with previous studies on preoperative clinics [10–12]. In clinical practice, the need for a preanesthetic oral examination was explained by a visiting staff member before the oral examination. However, at times, the patients did not even understand the anesthesia process. Insufficient health literacy and the use of dialect prevalent in Southern Taiwan increased the gap between the dentists and patients, indicating that patient-centered education is necessary in the future.

Strategies and policies focused on preventing perioperative dental injuries vary among institutions and countries. At some institutions, anesthesiologists evaluate the risks of dental trauma based on the dental history or a self-report questionnaire related to the patient’s dental status. Patients at high risk are transferred to dentists for a mouthguard [23–25]. Some Japanese institutions have included oral function management in an interdisciplinary system for perioperative management [26]. In Japan, treatment fees for perioperative oral management by dentists were included in the dental fee schedule of the National Health Insurance to prevent postoperative complications in 2012. Preoperative/perioperative oral management by dentists were proved to be effective in preventing the occurrence of postoperative aspiration pneumonia and reducing mortality and total medical costs [27–29]. Although anesthesiologists consistently work in the oral cavity of patients, they may not have studied the comprehensive education of teeth, surrounding tissues, and intraoral prostheses [9]. Therefore, the interdisciplinary cooperation involving anesthesiologists and dental team seems to be a better strategy to simultaneously reduce malpractice claims and improve patient safety and postoperative outcomes.

In the present study, although patients in the PAOE group were reported to have lower perceived service quality and patient satisfaction compared to the control group, the reported scores remained high. This indicates that the decline in service quality owing to the new program was limited. Future suggestions should focus on establishing adequate waiting times that are considered reasonable by patients. Patient-centered education related to the risk of perioperative dental injuries should be provided, and awareness of the oral condition for patient safety should be improved. Moreover, interprofessional education in continuing and undergraduate programs is necessary to improve professional quality.

This study has several limitations. First, owing to convenience sampling, a potential sample bias may exist. Second, only perceived service quality and satisfaction were evaluated, and the gaps between expectations and perceptions were not assessed. Future studies by addressing these limitations are warranted.

## Conclusions

Overall, patients in the PAOE group showed high satisfaction and perceived service quality. Although the implementation of an interdisciplinary program reduced perceived service quality in the dimensions of reliability and responsiveness, leading to lower patient satisfaction, its influence was limited. The interdisciplinary teamwork is a win–win strategy encouraged to improve patient safety and reduce malpractice claims.

### Electronic supplementary material

Below is the link to the electronic supplementary material.

Supplementary Material 1

## Data Availability

The datasets generated and/or analyzed during the current study are not publicly available because of the regulation of KMUHIRB, but are available from the corresponding author upon reasonable request.

## Abbreviations

PAOE: Preanesthetic oral examination
SERVQUAL: Service quality
M: Mean
SD: Standard deviation
CFI: Comparative fit index
TLI: Tucker-Lewis index
SRMR: Standardized root mean squared residual
RMSEA: Root mean square error of approximation
