# Supplementary material for: Patient perception of service quality to preanesthetic oral examination: a cross-sectional study using the SERVQUAL model
Source: BMC Oral Health. 2024 Jan 22;24:120. doi: 10.1186/s12903-024-03853-2 (PMC10801931; doi:10.1186/s12903-024-03853-2)
Supplement: Supplementary file 1 — Supplementary Material 1 [file 12903_2024_3853_MOESM1_ESM.docx]

Table 5 Comparing the perceived service quality by age group

| Variables | Age group (M±SD) | | | | | *P* | Post-hoc  comparison |
| --- | --- | --- | --- | --- | --- | --- | --- |
|  | 20-29 ^a^  (n=89) | 30-39 ^b^  (n=78) | 40-49 ^c^  (n=114) | 50-59 ^d^  (n=102) | 60-64 ^e^  (n=33) |  |  |
| Tangibles (4-28) | 25.93±3.08 | 25.01±2.80 | 25.49±2.98 | 24.59±3.99 | 25.55±3.16 | 0.063 |  |
| Reliability (5-35) | 32.53±4.00 | 31.85±3.35 | 32.03±4.05 | 31.22±4.86 | 32.24±3.86 | 0.288 |  |
| Responsiveness (4-28) | 25.74±4.31 | 24.67±4.83 | 22.94±7.18 | 21.26±7.28 | 21.87±7.22 | <0.001*** | a>c, d, e; b>d, e |
| Assurance (4-28) | 25.99±3.68 | 24.67±4.37 | 25.50±3.77 | 24.99±4.02 | 26.03±2.61 | 0.145 |  |
| Empathy (5-35) | 32.76±4.97 | 31.22±5.53 | 30.52±7.57 | 28.72±8.43 | 31.10±5.39 | 0.002** | a>c, d; b>d |

**: P<0.01; ***: P<0.001

Table 6 Comparing the perceived service quality by education level

| Variables | Education level (M±SD) | | | *P* | Post-hoc  comparison |
| --- | --- | --- | --- | --- | --- |
|  | College or higher ^a^  (n=256) | Senior/vocational high school ^b^  (n=129) | Junior high school or lower ^c^  (n=29) |  |  |
| Tangibles (4-28) | 25.47±2.89 | 24.96±3.43 | 24.88±5.48 | 0.337 |  |
| Reliability (5-35) | 32.22±3.62 | 31.72±4.12 | 30.11±7.03 | 0.183 |  |
| Responsiveness (4-28) | 24.70±5.21 | 21.19±7.66 | 21.42±8.06 | <0.001*** | a>b, c |
| Assurance (4-28) | 25.53±3.75 | 25.27±3.75 | 24.57±5.24 | 0.431 |  |
| Empathy (5-35) | 31.77±5.42 | 29.45±8.46 | 27.85±9.13 | 0.005** | a>b, c |

**: P<0.01; ***: P<0.001

Table 7 Comparing the perceived service quality by job status

| Variables | Education level (M±SD) | | | *P* | Post-hoc  comparison |
| --- | --- | --- | --- | --- | --- |
|  | Employed ^a^  (n=318) | Unemployed ^b^  (n=55) | Student ^c^  (n=26) |  |  |
| Tangibles (4-28) | 25.34±3.10 | 24.89±4.32 | 25.64±3.04 | 0.562 |  |
| Reliability (5-35) | 31.91±3.96 | 31.74±5.31 | 32.81±3.43 | 0.526 |  |
| Responsiveness (4-28) | 23.22±6.61 | 23.78±6.41 | 26.16±2.43 | <0.001*** | c>a |
| Assurance (4-28) | 25.43±3.75 | 25.22±4.09 | 26.00±2.93 | 0.679 |  |
| Empathy (5-35) | 30.70±6.90 | 30.24±7.56 | 33.50±2.67 | <0.001*** | c>a, b |

***: P<0.001
